# Maternal Diet and Infant Risk of Eczema and Food Allergy: A Systematic Review

**DOI:** 10.7759/cureus.45114

**Published:** 2023-09-12

**Authors:** Faten Sid Idris, Humna Anis Shaikh, Ilma Vahora, Kiran Prasad Moparthi, Majdah T Al Rushaidi, MeghanaReddy Muddam, Omobolanle A Obajeun, Abdelrahman Abaza, Arturo P Jaramillo, Pousette Hamid

**Affiliations:** 1 Pediatrics, California Institute of Behavioral Neurosciences & Psychology, Fairfield, USA; 2 General Surgery, Saint George's University School of Medicine, Chicago, USA; 3 General Surgery, California Institute of Behavioral Neurosciences & Psychology, Fairfield, USA; 4 Medicine, SVS Medical College, Mahabubnagar, IND; 5 Medicine, California Institute of Behavioral Neurosciences & Psychology, Fairfield, USA; 6 Psychology, California Institute of Behavioral Neurosciences & Psychology, Fairfield, USA; 7 Medicine, SVS Medical College, Hyderabad, IND; 8 Pediatrics, Al Zahra Private Hospital, Dubai, ARE; 9 Pathology, California Institute of Behavioral Neurosciences & Psychology, Fairfield, USA; 10 General Practice, California Institute of Behavioral Neurosciences & Psychology, Fairfield, USA; 11 Neurology, California Institute of Behavioral Neurosciences & Psychology, Fairfield, USA

**Keywords:** infant food intolerance, food hypersensitivity, infant food allergy, atopic dermatitis, skin allergy, noncontagious skin inflammation, eczema, child*, diet during pregnancy, maternal alimentation

## Abstract

A significant portion of the pediatric population is affected by allergy diseases, which have become a worldwide public health concern. Could maternal diet during pregnancy or breastfeeding influence allergy outcomes in offspring? If this cause-and-effect relationship exists, it will be simpler to design prevention strategies to reduce the incidence of allergic disorders in children, reduce costs to the public health system and to parents, and improve the quality of life of allergic children and their parents.

In this systematic review, we will visit the literature from January 2019 to December 2022 to see if any relationship was found between maternal nutrition and its consequences on children’s allergy occurrence. We will focus only on food allergy and eczema outcomes in the offspring. Also, we will summarize what was found to be protective or nonprotective to better control the outcomes if applied in the future.

## Introduction and background

Does maternal alimentation influence the development of allergy diseases in offspring?

Nutritional factors are among the environmental elements that can influence an individual’s acquisition of chronic non-communicable diseases such as allergies [1]. Allergy is viewed as the initial sign of immune system dysfunction and tolerance impairment [2]. Maternal genes and environmental factors mold organogenesis and shape the fetal immune system. This notion stems from the theory that the root cause of most diseases and their risk factors emanate from tiny disruptions during organogenesis and rapid cell division [3]. Therefore, exposure to food allergens during pregnancy and the first 1000 days of life eventually affects the immune system’s development [4]. Immune responses to allergy diseases stimulate lymphocyte-mediated responses, specifically T lymphocyte helper 2 (TH-2) activity, promoting immunoglobulin E and eosinophile synthesis. T lymphocyte helper 1 (TH-1), produced secondary to infection, downregulates TH-2 production; Th-2 by secreting interleukin 4 (IL-4) downregulates Th-1 activity; and T lymphocyte regulators (T-regs) suppress both Th-1 and Th-2. Overactivity of Th-2 or a lack of regulation by Th-1 or T-regs probably increases the likelihood of developing allergy diseases [4]. Food allergy and eczema are highly connected and infant eczema is still poorly comprehended but considered to be controlled by environmental and genetic factors [5-6].

For the last 50 years, food allergies have become a worldwide problem [4], and over the last 30 years, eczema prevalence has increased significantly and has become a nationwide problem [7]. Food allergy has an important effect on children/caregivers life quality [8,9], and it is a potential cause of serious anaphylaxis reactions that can be fatal [4] with huge costs for the healthcare system and for parents [10]. Eczema and food allergy are strongly linked; among people with severe eczema one-third have documented food allergy [5-11], resulting in serious psychosocial and behavioral problems [12]. Comprehending how early diet affects offspring immune system development [13] may help initiate prevention strategies to minimize the morbidity and costs of food allergies and atopic dermatitis. In this systematic review, we will be reviewing the last five years (2019-2023) of literature to see if there is any evidence linking maternal diet and infant risks of developing eczema and food allergy and to search for any preventive procedure that was elucidated and is applicable. We will focus only on eczema and food allergy, and we will not consider in this review other allergy diseases such as asthma, or allergic rhinitis. The goal of this study is to find out if it is true that maternal alimentation influences allergic outcomes in offspring and if we can prevent it.

## Review

Methods

We conducted our systematic review based on the recommendation of the Preferred Reporting Items for Systematic Reviews and Meta-Analysis (PRISMA) 2020. We used the online databases PubMed and Google Scholar between May 8-31, 2023. The search was conducted by search of relevant studies using regular keywords and Medical Subject Headings (MeSH). The search strategy is summarized in Table 1. Keywords were used in different combinations using Boolean operators like “AND” and “OR".

**Table 1:** The search strategy MeSH: Medical Subject Headings

Regular/MeSh keywords	Database	Result number	
Maternal alimentation (( "Maternal Nutritional Physiological Phenomena/ethnology"[Majr] OR "Maternal Nutritional Physiological Phenomena/genetics"[Majr] OR "Maternal Nutritional Physiological Phenomena/immunology"[Majr] OR "Maternal Nutritional Physiological Phenomena/physiology"[Majr] )) OR ( "Maternal Nutritional Physiological Phenomena/ethnology"[Majr:NoExp] OR "Maternal Nutritional Physiological Phenomena/genetics"[Majr:NoExp] OR "Maternal Nutritional Physiological Phenomena/immunology"[Majr:NoExp] OR "Maternal Nutritional Physiological Phenomena/physiology"[Majr:NoExp] )	Pub Med	886	
Child* ("Mother-Child Relations"[Majr]) OR "Mother-Child Relations"[Majr:NoExp]	Pub Med	11690	
Eczema (( "Eczema/congenital"[Majr] OR "Eczema/diagnosis"[Majr] OR "Eczema/diet therapy"[Majr] OR "Eczema/enzymology"[Majr] OR "Eczema/etiology"[Majr] OR "Eczema/genetics"[Majr] OR "Eczema/immunology"[Majr] OR "Eczema/nursing"[Majr] )) OR ( "Eczema/congenital"[Majr:NoExp] OR "Eczema/diagnosis"[Majr:NoExp] OR "Eczema/diet therapy"[Majr:NoExp] OR "Eczema/enzymology"[Majr:NoExp] OR "Eczema/etiology"[Majr:NoExp] OR "Eczema/genetics"[Majr:NoExp] OR "Eczema/immunology"[Majr:NoExp] OR "Eczema/nursing"[Majr:NoExp]	Pub Med	2190	
Food Allergy (( "Food Hypersensitivity/classification"[Majr] OR "Food Hypersensitivity/congenital"[Majr] OR "Food Hypersensitivity/diagnosis"[Majr] OR "Food Hypersensitivity/diet therapy"[Majr] OR "Food Hypersensitivity/ethnology"[Majr] OR "Food Hypersensitivity/etiology"[Majr] OR "Food Hypersensitivity/genetics"[Majr] OR "Food Hypersensitivity/immunology"[Majr] OR "Food Hypersensitivity/nursing"[Majr] OR "Food Hypersensitivity/prevention and control"[Majr] )) OR ( "Food Hypersensitivity/classification"[Majr:NoExp] OR "Food Hypersensitivity/congenital"[Majr:NoExp] OR "Food Hypersensitivity/diagnosis"[Majr:NoExp] OR "Food Hypersensitivity/diet therapy"[Majr:NoExp] OR "Food Hypersensitivity/ethnology"[Majr:NoExp] OR "Food Hypersensitivity/etiology"[Majr:NoExp] OR "Food Hypersensitivity/genetics"[Majr:NoExp] OR "Food Hypersensitivity/immunology"[Majr:NoExp] OR "Food Hypersensitivity/nursing"[Majr:NoExp] OR "Food Hypersensitivity/prevention and control"[Majr:NoExp] )	Pub Med	10226	
Complete search strategy Maternal alimentation AND epigenetic environment AND Diet during pregnancy OR eating habits during pregnancy OR proteins consumption during pregnancy OR food during pregnancy AND Child*AND Eczema OR atopic dermatitis OR skin allergy OR noncontiguous chronic skin inflammation Condition AND Infant food allergy OR infant protein consumption and sensitivity OR food hypersensitivity OR Infant food intolerance AND (( "Maternal Nutritional Physiological Phenomena/ethnology"[Majr] OR "Maternal Nutritional Physiological Phenomena/genetics"[Majr] OR "Maternal Nutritional Physiological Phenomena/immunology"[Majr] OR "Maternal Nutritional Physiological Phenomena/physiology"[Majr] )) OR ( "Maternal Nutritional Physiological Phenomena/ethnology"[Majr:NoExp] OR "Maternal Nutritional Physiological Phenomena/genetics"[Majr:NoExp] OR "Maternal Nutritional Physiological Phenomena/immunology"[Majr:NoExp] OR "Maternal Nutritional Physiological Phenomena/physiology"[Majr:NoExp] ) AND ("Mother-Child Relations"[Majr]) OR "Mother-Child Relations"[Majr:NoExp] AND (( "Eczema/congenital"[Majr] OR "Eczema/diagnosis"[Majr] OR "Eczema/diet therapy"[Majr] OR "Eczema/enzymology"[Majr] OR "Eczema/etiology"[Majr] OR "Eczema/genetics"[Majr] OR "Eczema/immunology"[Majr] OR "Eczema/nursing"[Majr] )) OR ( "Eczema/congenital"[Majr:NoExp] OR "Eczema/diagnosis"[Majr:NoExp] OR "Eczema/diet therapy"[Majr:NoExp] OR "Eczema/enzymology"[Majr:NoExp] OR "Eczema/etiology"[Majr:NoExp] OR "Eczema/genetics"[Majr:NoExp] OR "Eczema/immunology"[Majr:NoExp] OR "Eczema/nursing"[Majr:NoExp] ) AND(( "Food Hypersensitivity/classification"[Majr] OR "Food Hypersensitivity/congenital"[Majr] OR "Food Hypersensitivity/diagnosis"[Majr] OR "Food Hypersensitivity/diet therapy"[Majr] OR "Food Hypersensitivity/ethnology"[Majr] OR "Food Hypersensitivity/etiology"[Majr] OR "Food Hypersensitivity/genetics"[Majr] OR "Food Hypersensitivity/immunology"[Majr] OR "Food Hypersensitivity/nursing"[Majr] OR "Food Hypersensitivity/prevention and control"[Majr] )) OR ( "Food Hypersensitivity/classification"[Majr:NoExp] OR "Food Hypersensitivity/congenital"[Majr:NoExp] OR "Food Hypersensitivity/diagnosis"[Majr:NoExp] OR "Food Hypersensitivity/diet therapy"[Majr:NoExp] OR "Food Hypersensitivity/ethnology"[Majr:NoExp] OR "Food Hypersensitivity/etiology"[Majr:NoExp] OR "Food Hypersensitivity/genetics"[Majr:NoExp] OR "Food Hypersensitivity/immunology"[Majr:NoExp] OR "Food Hypersensitivity/nursing"[Majr:NoExp] OR "Food Hypersensitivity/prevention and control"[Majr:NoExp] )	Pub Med	8843	
Maternal alimentation AND child* AND eczema AND food allergy	Google Scholar	17,700	

Inclusion and Exclusion Criteria

We selected peer-reviewed articles from January 2019 to December 2023 published in the English language. Six duplicates were found and deleted after the application of inclusion/exclusion criteria and screening. We excluded gray literature and animal studies. Our study included only human studies in the category of systematic review, review, and cohort studies.

Quality Assessment Tools

The measurement tools used to assess our systematic review were the quality assessment for systematic review A MeaSurement Tool to Assess systematic Reviews (AMSTAR), and a scale for the assessment of the non-systematic review article Scale for the Assessment of Narrative Review Articles (SANRA) checklist, and the New Castle Ottawa tool for cohort studies. Each assessment tool has its own scoring system. Studies that received a score of more than 70% on each evaluation tool were selected for inclusion in this systematic review; we excluded studies that were of low quality. Table 2 outlines the quality assessment results of the final articles included in this systematic review.

**Table 2:** Quality assessment of the articles included in this systematic review AMSTAR: A MeaSurement Tool to Assess systematic Review SANRA: Scale for the Assessment of Narrative Review Articles

Studies	Type of studies	Critical appraisal tool	Total score found	Accepted score
Vale et al. [14]. Donovan et al. [15]. Venter et al. [16]	Systematic review	AMSTAR	12-13	9-11
Acevedo et al. [1]. Warner et al. [3]. Fujimura et al. [4]. Di Costanzo et al. [13]. Jiao et al. [17]. Danielewicz et al. [18]	Review	SANRA	75%-83%	≥70%
Pretorius et al. [19]. Zeng et al. [20]. Brzozowska et al. [21]	Cohort	NEWCASTLE - OTTAWA	7-8	≥7

Results

**Figure 1:**
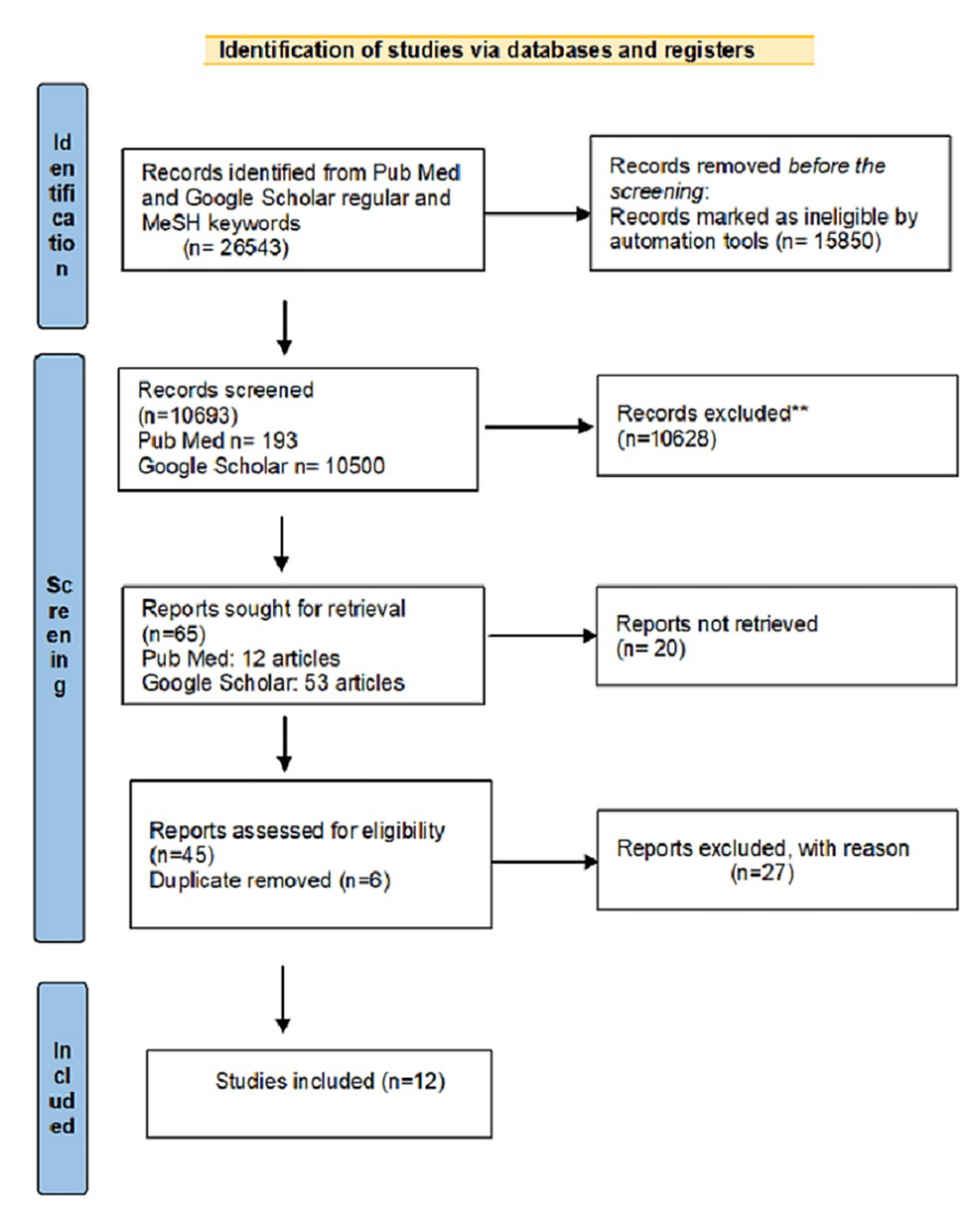
Preferred Reporting Items for Systematic Reviews and Meta-Analysis (PRISMA) flow diagram for the search strategy

We selected 65 studies after screening the titles and reviewing them. Six duplicates were found and removed, and 45 studies were selected for eligibility assessment. We excluded seven abstracts, four review articles, three observational studies, two clinical trials, 11 studies without a clear method section, and four traditional reviews were excluded. We finally included three systematic review studies, three cohort studies, and six review studies. Figure 1 displays the PRISMA flow diagram.

Data Collection

Data included in this systematic review is resumed in Table 3 below. The table provides a summary of the study characteristics including the authors' names, type of study conducted, the year of the studies, the journal/book name where the study was published, the purpose, and the outcomes of the studies.

**Table 3:** Table of study characteristics n-3/n-6: Omega three polyunsaturated fatty acids/ omega six polyunsaturated fatty acids

Study	Authors	Type of study	Year of study	Journal/book	Purpose of the study	Conclusion
1	Acevedo et al. [1]	Review	2021	Nutrients	Find if epigenetic modification and prenatal/ postnatal alimentation contribute to children’s allergy development.	Gestational deficiency of vitamin D is associated with the risk of eczema in offspring.
2	Warner et al. [3]	Review	2022	Nutrients	Factors that influence allergy development in offspring during neonatal life.	Low cord blood n-3/n-6 ratios were linked to a higher frequency of infant eczema.
3	Fijimura et al. [4]	Review	2019	Frontiers Immunology	Identify maternal characteristics that affect offspring allergy risk during pregnancy, and breastfeeding.	High maternal consumption of cow milk, peanut/ tree nut during pregnancy, and breastfeeding decrease allergy risk in offspring.
4	Di Costanzo et al. [13]	Review	2022	Nutrients	How nutrition at early age of life influences immune system development and allergy outcomes in offspring and role of epigenetic factors.	Omega 3 supplementation during pregnancy may confer a protective effect against eczema in offspring.
5	Jiao et al. [17] 2022	Review	2022	Front Immuno	Comprehend the maternal influence in offspring allergy development is essential to implement prevention measures.	Prenatal maternal consumption of peanut and tree nut (P/TN) was linked to a low-risk allergy to P/TN and the same observations were reported during the breastfeeding period in offspring.
6	Danielewicz et al. [18]	Review	2022	Nutrients	Describe the factor that affects the breastfeeding period, and allergy development in offspring.	cow’s milk formula in breast fed infant was found to increase allergy to cow milk and other food allergy if introduced in the first 3 days of life and the presence of high-risk allergy to cow milk in breastfed infants who didn’t receive cow’s milk formula between 1 and 2 months of life.
7	Vale et al. [14]	Systematic Review	2021	Nutrients	Establish recommendations on food allergy prevention for a professional use.	No maternal dietary exclusion or restriction during pregnancy and breastfeeding is indicated for allergy prevention.
8	Pretorius et al. [19]	Cohort Study	2019	Nutrients	Analyze the relationship between maternal ingestion of fiber and allergy disease risk.	High maternal ingestion of resistant starch in late pregnancy was associated with a high risk of eczema in offspring.
9	Donovan et al. [15]	Systematic Review	2020	USDA Nutrition Evidence Systematic Review	Assess a link between maternal diet during pregnancy and lactation and offspring risk of atopic disease and food allergy.	Pregnant women’s consumption of Peanut and tree nut play has no allergy risk in offspring.
10	Zeng et al. [20]	Cohort study	2021	Frontiers Nutritions	Investigate the potential maternal consumption of protein during pregnancy and eczema risk in offspring.	High maternal ingestion of poultry protein during pregnancy was associated with an increased risk of eczema in offspring.
11	Venter et al. [16]	Systematic Review	2022	Allergy	Create a new maternal diet index during pregnancy that could be linked to the offspring risk of allergy.	Mother consumption of Yogurt and vegetables showed decreased allergy in offspring.
12	Brzozowska et al. [21]	Cohort study	2022	Environmental Research	Analyze the maternal type of diet and allergy risk in offspring.	Vitamin E in pregnant women was associated with a high risk of eczema in offspring.

Discussion

Pre-natal Risk of Allergen Sensitization

During the first 1000 days of life, factors related to environment and nutrition could impact the risk of food allergy diseases and the immune system’s development via interaction between epigenetic factors and individual genetic predisposition [1-13]. It is well known that maternal immunoglobulin G (IgG) is the only Ig that can pass through the placental membrane and provide passive immunity to the fetus, which is modulated by the neonatal Fc receptor (FcRn) present in the placental barrier [22-23]. For instance, infection during pregnancy influences the offspring's immunity through the activity of Th1/Th2 cytokines and the fetal-maternal immune transfer [17].

There are four subtypes of IgG (IgG1, IgG2, IgG3 and IgG4) and Ig4 is closely associated with immunoglobulin E (IgE) secretion during allergy reactions. Individuals with allergy tolerance have much greater allergen-specific IgG4/IgE ratios than do those with allergy-prone [24]. The reasoning for this finding is that non-atopic mothers fabricate high levels of IgG1 and IgG3 antibodies that cross the placenta and confer protection to the fetus, whereas atopic mothers produce high levels of IgE and IgG4 antibodies that cannot cross the placental barrier [17]. The newborn may be predisposed for allergy sensitization and show early allergy symptoms if fetal IgE is improperly suppressed by the low maternal production of IgG1 and IgG3 [17]. Even though the crucial role of maternal allergen transmission and offspring risk of developing allergies is not fully understood [25], it is accepted that an immune complex made up of IgG and an allergen can be transferred from the mother to the fetus [25]. Thus, fetal exposure to these antigens in the uterine environment may promote tolerance to these particular food sources throughout the newborn period [17].

Amniotic liquid comprises maternal decayed TH-2 cells, antigens and allergens to which she was exposed to. As the fluid is swallowed by the fetus, it creates a possible pathway for prenatal TH-2 sensitization [26-27]. This notion is reinforced by the correlation between mother exposure to house dust, mites and cat allergens during pregnancy and cord blood IgE levels found [28]. The risk of fetal sensitization to allergens depends on the level of allergens; very low levels are unable to generate sensitization, whereas very high levels of allergens promote tolerance via a different process [3]. At 16 weeks of gestation, amniotic fluid contains 10% of maternal IgE, and fetal lamina propria contains IgE receptors; thus, there is an opportunity for antigen-focusing manifestation. With low IgE levels and high affinity for IgE receptors, sensitization is observed 100-1000 times less concentration of allergen than without the presence of IgE antibodies [3-29].

Primary allergen sensitization takes place via fetal intestinal mucosa during the second trimester of pregnancy and via fetal circulation in the form of IgG-antigen complexes that traverse the placenta at the third trimester [3]. High maternal exposure to a specific allergen induces consequently high IgG levels for those allergens, which lessen the probability of allergen sensitization in offspring; this was documented for egg, cat, and dog allergens [30-31]. Another example reported about ongoing rye-grass immunotherapy during pregnancy correlated to decreased rye-grass allergy in offspring [32].

Breast Feeding and Risk of Allergen Sensitization

Breastfeeding has been the natural method to feed newborns and infants [18]. The World Health Organization (WHO) recommends using human milk as a child's only source of nutrition for the first six months of life, followed by the introduction of complementary foods until the child is two years old because it may protect against eczema and food allergies [33-34]. Maternal milk has immediate benefits during the neonatal period but also provides long-term benefits as it protects against overweight and diabetes, similarly, it protects from infection in early childhood [18].

Human colostrum contains 90% of secretory immunoglobulin A (IgA) that covers the infant’s mucosal surface and supports newborn immunity system maturation until the infant starts producing its own IgA by increasing immunoglobulin A and probiotics [17]. It is known that breast milk in nonatopic mothers provides protection against food allergy [17] while infants of atopic mothers may present a higher risk of acquiring allergies [35], due to the high level of cytokines and chemokines and low level of transforming growth factor (TGF)-b1 in their breast milk. A proper amount of TGF-b1 in maternal milk enhances tolerance against numerous food allergens transmitted to the newborn intestinal mucosa via breastfeeding, solids, and formula [17].

Maternal Diet and Food Allergy in Offspring

Fujimura et al. [4] reported that food allergen consumption during pregnancy protects against allergy sensitization in children. A Finland study pointed out that non-allergic mothers with high milk intake during pregnancy were linked to a lower chance of cow’s milk allergy in their offspring which demonstrated tolerance reactions in utero [4-36]. Another prospective study showed a low prevalence of peanut or tree nut allergy in children of non-allergic mothers who had at least five servings of peanuts or tree nuts a week during pregnancy, but no preventive effect was found in children of allergic mothers to peanuts and tree nuts [37]. A cohort study in Canada that studied the relationship between breastfeeding and maternal peanut consumption found that mothers who consumed peanuts while breastfeeding, mothers who delayed peanut introduction to their babies until after one year old [38], or mothers who avoided consuming peanuts during breastfeeding and introduced peanut to their babies at 12 months, had a higher chance of peanut sensitization. These findings imply that maternal peanut ingestion while breastfeeding and child exposure to peanuts throughout the first year, both conditions present, offer protection against peanut allergy [4].

According to Jiao et al. [17], American Academy of Pediatrics in 2000 instructed allergic pregnant and breastfeeding mothers and their offspring to avoid peanut ingestion [39]. However, in 2008, they canceled this recommendation [40]. Recent studies showed that tree nut and peanut consumption during pregnancy had no allergic outcomes in offspring and might even play a preventive role [41]. Moreover, studies showed high prenatal consumption of peanut and tree nut (P/TN) was linked to a low risk of allergy to P/TN, and the same observations were reported during the breastfeeding period [37].

Danielewicz [18] pointed out the potential effect of cow's milk intake and allergy outcomes in breastfed infants. One study showed that early exposure (the first three days of life) to cow’s milk formula in breastfed infants was found to increase allergy to cow’s milk and to other food allergies. From that, we can infer that the first days of life could play a crucial role in allergy development. In this trial, newborns received either breast milk and cow’s milk formula or breast milk and hydrolyzed formula as a supplement. Sensitization to cow's milk, other food allergies and anaphylaxis were estimated in the second year of life [42]. Another study compared babies fed with no cow's milk formula with babies fed at least 10 ml of cow's milk formula between one and two months of age. They found a high risk for allergies at the age of six months in the babies who didn’t receive cow's milk formula [43]. In both trials, infants that did not receive cow's milk formula had a hydrolyzed formula in the first study and soy formula in the second study [18]. Two studies show conflicting results; this conflict could be due to the timing of cow's milk introduction or due to the formula type used (soy formula/amino acid formula).

The WHO defined a set of 28 guidelines as "any document that contains recommendation for clinical health practice or public health policy" [44]. Vale et al.'s [14] systematic review offered a comparison between the preventive guidelines for food allergies. All 28 guidelines suggest no maternal dietary exclusion or restriction during pregnancy, and breastfeeding is indicated for allergy prevention [14].

Di Costanzo et al. [13] mentioned that omega-three polyunsaturated fatty acids (n-three PUFAs) alleviate allergy symptoms and provide anti-inflammatory action [45], whereas omega-six polyunsaturated fatty acids (n-six PUFAs) are pro-inflammatory and facilitate the Th2 immune response and consequently allergy disease development [46]. High intake of n-three long-chain (LC) PUFAs during pregnancy plus docosahexaenoic acid (DHA) oil was associated with less risk of IgE-mediated atopic dermatitis and decreased sensitization to hens’ eggs when compared to the offspring of women not supplemented during pregnancy [47]. Research conducted on pregnant and lactating women showed that n-three LCPUFAs supplementation (combination of DHA and eicosapentaenoic acid (EPA)) promotes good effects on the immune system [48]. The effects of n-three LCPUFAs on the immune system are still not fully comprehended, but recent studies imply that epigenetic mechanisms may be involved [13]. This concept is supported by Lee et al., who report that providing mothers with n-three PUFA during pregnancy may impact DNA methylation levels of genes encoding Th1 and Th2 cytokines (IFN-γ and IL-13, respectively) and as a result, influence the Th1/Th2 balance in infants [49].

Maternal Diet and Offspring Risk of Eczema

Pretorius et al.'s cohort study approved by the Princess Margaret Hospital Human Research Ethics Committee (HREC approval number 1942EP) focused on maternal fiber intake and risk of allergy. High maternal ingestion of resistant starch after 36 weeks of pregnancy resulted in a high risk of development of eczema in offspring without allergen sensitization. A limitation of this study was that resistant starch was not subdivided into the subtypes of resistant starch (RS1, RS2, RS3, RS4) with different functions and structures whose consequences on infant immune system development demand more studies [19].

Donovan et al. conducted a systematic review to look for a link between pregnant women’s diet and the risk of allergy and atopic disease in children. Different scenarios of consumption were explored, no mother intake vs/child intake only vs/mother intake only vs/mother and child intake showed that mother and child intake vs/no intake was correlated to a decreased risk of eczema at 18 months in children [15]. 

Acevedo et al. [1] explained that vitamins A and D play a crucial role in the function of the immune system and allergy disease development, and epigenetic mechanisms are known to influence the relationship between vitamin and allergy development. For example, retinoid acid (RA) (vitamin A metabolite) has a central role during development by regulating transcription, and it has been demonstrated that the RA signaling pathway inhibits the transcriptional and epigenetic programs by activating TH9 (secreted by IL4, Th2, TGB) that plays an important role in atopy. Furthermore, RA halts IgE class switching recombination by blocking histone acetylation in the gene promoter region and consequently blocks gene expression [50]. In addition, evidence has shown that the lack of vitamin D during pregnancy reduces TH1/TH2 and IF-Y secretion and raises IL-4 secretion [51]. Thus, vitamin D deficiency is associated with increased DNA methylation activity and also increased IF-Y methylation activity (IF-Y is known to block TH2 cell production). This results in low gene expression or gene silencing [51].

A gestational deficiency of vitamin D is associated with a higher risk of eczema, partly caused by a diminished DNA methylation of a gene involved in producing reactive oxygen species. This can be reversed by vitamin D supplementation during pregnancy [52].

Zeng et al.'s prospective cohort study examined the potential association between maternal dietary protein consumption during pregnancy and infant eczema risk. Four kinds of protein were determined: proteins found in red meat, fish, poultry, plants, dairy, and eggs. Eczema risk was found to be higher in the group with high poultry consumption compared with the other dietary protein patterns, and a lower incidence of infant eczema at six months was found [20]. This outcome was consistent with a prior Australian study showing a positive correlation between maternal pre-conception poultry ingestion and offspring atopic dermatitis [53].

Warner et al. stated that the Western diet is poor in n-three PUFAs and rich in n-six PUAFs [3]. This pattern of diet was associated with a high incidence of allergic diseases [49]. Low cord blood n-three: n-six ratios were linked to a higher frequency of infant eczema due to high maternal meat consumption and less consumption of fish, which contains high levels of n-three PUFAs [54]. However, giving fish oil supplements to mothers during pregnancy and lactation was related to inconsistent results in offspring [3]. 

Venter et al.'s study objective was to create a new index of maternal diet during pregnancy that would be linked to allergy manifestation in offspring. Forty-one separate variables were used to evaluate the association of each food propensity questionnaire item with any allergy manifestation, excluding wheeze. Yogurt and vegetables showed allergy protective effects, while rice or grains, french fries/fried potatoes, red meats, fruit juice that is 100% pure, and cold cereal were related to negative outcomes and increased disease. Every one-unit rise in the maternal diet index was associated with a 23% probability reduction for eczema. There was no correlation between diet index and food allergies identified in this study (only 3% of food allergies were found) [16].

Based on Brozozowska et al.'s study, improper intake of vitamin E in pregnant women was associated with an increased risk of eczema in children aged between two and seven years. In addition, they found a decreased risk of eczema in mothers who consumed unhealthy food during pregnancy [21]. 

Analysis

We conducted this systematic review to find out about any relation between maternal alimentation and offspring risk of food allergy and eczema. We could infer from our search that the offspring's immune system development and sensitization to allergens are deeply linked to the mother’s allergen exposure during pregnancy and breastfeeding. There is agreement regarding food restriction during pregnancy and breastfeeding being associated with an increased risk of allergy outcomes in offspring. Also, it seems that high consumption of cow milk and peanuts/tree nuts during pregnancy plays a protective role in food allergy disease in offspring. In addition, yogurt and probiotic consumption during pregnancy confer a protective effect against eczema in infants, whereas mothers who consumed high levels of resistant starch and poultry protein during pregnancy had increased odds of eczema in their offspring. In recent studies, we identified factors as protective, potentially protective, or non-protective in offspring allergy outcomes. Table 4 summarizes our findings.

**Table 4:** Mother's food allergen consumption and children risk of allergy disease protection status

Protective factors	Potentially protective factors	Non-protective factors
High cow's milk consumption	Vitamin A	Resistant starch
High ingestion of peanut/tree nut, yogurt, vegetables	Vitamin D, Vitamin E, fish oil	High ingestion of poultry protein, red meat, rice, grain, and fried potatoes

It is clear that mother-to-offspring exposure during pregnancy to allergens plays a crucial role in the offspring's immune system tolerance development. The question that arises may be the focus of future study: is there anything to be done for an allergic mother to minimize allergy outcomes in her offspring? Could allergen immunotherapy for allergic mothers when they are pregnant and/or breastfeeding be a solution? To find out a randomized clinical trial must be conducted. Is there a possibility to implement protection approaches that aim to mitigate allergy outcomes in offspring with the information we already have?

We could mention some limitations of this systematic review, like using recent literature from 2019 to 2022, some of the articles included in this systematic review had references dated back before 2019. Also, we focused only on eczema and food allergy outcomes in offspring; we did not approach other outcomes like asthma and allergic rhinitis. Some of the studies we visited evaluated allergy risk in children older than two years of age. We took into consideration only clear findings about mother food aliment's consumption and risk effects or protective effects on allergy development in offspring. 

## Conclusions

We started this systematic review by asking a question about the presence of any link between pregnant women’s diet and allergy disease development in offspring. There is a clear association between maternal exposure to food allergens during pregnancy and infant allergen sensitization. Literature agrees that food avoidance during pregnancy induces a high allergy risk in children. For instance, we found that high maternal intake during pregnancy of cow's milk and peanuts/tree nuts reduces offspring’s allergy food to these components, whereas mother consumption of poultry protein and resistant starch during pregnancy could have negative outcomes and a high likelihood of allergy development in offspring. We can infer from the correlation between the literature findings that maternal diet may have a significant impact on the offspring's allergy status. Will it be possible to set preventative measures into action or should we wait for more evidence?
